# Psychological Interventions for Pregnant Women in Chemical, Biological, Radiological, and Nuclear Incidents: A Systematic Review

**DOI:** 10.1002/hsr2.72217

**Published:** 2026-06-02

**Authors:** Maryam Shabany, Zahra Chegeni, Simintaj Sharififar, Fatemeh Teymouri, Maryam Azizi

**Affiliations:** ^1^ Community Health and Research Department, School of Nursing Aja University of Medical Sciences Tehran Iran; ^2^ Health in Disasters and Emergencies Department, School of Nursing Aja University of Medical Sciences Tehran Iran; ^3^ Student Research Committee, School of Nursing Aja University of Medical Sciences Tehran Iran

**Keywords:** CBRN, disasters, pregnant women, psychological intervention

## Abstract

**Background and Aims:**

Chemical, Biological, Radiological, and Nuclear (CBRN) incidents increasingly threaten public health, especially with the rise of unconventional weapons. Pregnant women are particularly vulnerable to psychological impacts. Psychological First Aid (PFA) is a key supportive intervention. This study explores current psychological strategies and aims to improve mental health outcomes for pregnant women in CBRN emergencies.

**Methods:**

In this systematic review, four electronic databases—PubMed, Embase, Web of Science, and Scopus—were initially searched up to November 4, 2023 and subsequently updated in July 2025; no additional eligible studies were identified in the updated search. The review followed the Preferred Reporting Items for Systematic Reviews and Meta‐Analyses (PRISMA) guidelines. A total of 7228 records were identified. After removing 2633 duplicates, 4595 records remained for screening. Following title and abstract screening, 103 full‐text articles were assessed for eligibility, of which 25 studies met the inclusion criteria and were included in the final analysis.

**Results:**

This study emphasized the importance of psychological responses and support during CBRN incidents, especially for pregnant women. Interventions at individual, organizational, and social levels may help reduce psychological distress. Individually, these include PFA, counseling, and stress management. Organizational interventions involve planning mental health services and necessary support in hospitals and medical centers. Socially, they include promoting support networks and helping pregnant women cope with incidents.

**Conclusion:**

This review emphasized that in CBRN incidents, supporting the mental health of pregnant women at individual, organizational, and social levels was critical. The study supported a three‐pronged intervention framework aiming to reduce the psychological impact of CBRN incidents and empower individuals and communities to rebuild and thrive afterward. These results emphasized that joint efforts of healthcare providers, policymakers, and community organizations were essential to provide psychosocial care that is comprehensive, accessible, and sensitive to the distinct needs of affected individuals.

## 1 Background

In recent years, advancements in technology and the growing inclination of terrorist groups toward unconventional weapons have heightened the risk and consequences of chemical, biological, radiological, and nuclear (CBRN) incidents [1, 2, 3]. These incidents present a major threat to society, as CBRN agents are highly dangerous and challenging to control due to their covert nature and unpredictable effects. In some cases, contamination may result in delayed clinical symptoms, further complicating response efforts [4]. The intentional use of CBRN agents exacerbates the situation, increasing concerns among medical and health services [5, 6, 7, 8].

Unlike incidents such as flooding, where the affected area and damages can be quantified, CBRN incidents present significant challenges in estimating casualties, financial losses, and psychological or social consequences [9]. In addition, the health consequences of exposure may not be immediately visible, potentially leading to long‐term effects on future generations, both physically and mentally [10].

Disaster response efforts typically focus on addressing physical damage, often overlooking the emotional and psychological distress experienced by affected individuals. This oversight can contribute to heightened psychological distress and an increased risk of psychiatric disorders, including substance abuse, domestic violence, stress, and anxiety in the aftermath of major disasters [11]. Conditions such as acute stress disorder (ASD) and posttraumatic stress disorder (PTSD) are commonly associated with trauma and can be associated with major depression, substance abuse, family conflicts, and generalized anxiety disorder [10, 12]. Fear of the future and uncertainty surrounding the consequences of CBRN incidents make it difficult for the public to understand and follow appropriate protective measures [13].

Pregnant women, in particular, represent a vulnerable group during disasters. Pregnancy involves a complex interplay of physiological, hormonal, and emotional changes, which make expectant mothers more susceptible to emotional distress [14, 15]. Pregnant women exposed to traumatic events, including CBRN incidents, are at heightened risk for developing PTSD and depression, which can adversely affect both maternal and fetal health [14]. Although no current studies have specifically examined the psychological impact of CBRN incidents on pregnant women, extensive research from other types of disasters provides valuable insights. For instance, Giarratano et al. (2019) [16] documented persistent anxiety, depression, and post‐traumatic stress disorder (PTSD) among pregnant women even years after recovery from natural disasters. Similarly, research during the COVID‐19 pandemic has consistently reported elevated levels of mental health symptoms such as anxiety and depression among expectant mothers [17]. These findings underscore the heightened psychosocial vulnerability of pregnant women in the aftermath of any large‐scale emergency. However, there remains a critical gap in the literature concerning the specific psychological effects of CBRN events on this vulnerable population [17, 18].

Uncertainty about the physical and psychological effects of CBRN incidents can cause frustration and emotional exhaustion, particularly among displaced populations. This distress is often intensified by mass media and social networks through misinformation, rumors, and stigmatization [19, 20, 21]. Given the psychological shock of terrorism‐related events, it is crucial to address psychosocial impacts among vulnerable groups [22].

Sudden outbreaks of public health events consistently challenge mental health service systems. The lack of psychosocial support frameworks and trained mental health professionals increases the risk of psychological distress and social depression in at‐risk communities [23, 24, 25, 26]. Often, these consequences are overlooked, highlighting the need for improved mental health infrastructure and support [24, 26, 27]. For instance, the Chernobyl Forum identified mental health concerns as the most critical public health issue following the 1986 nuclear disaster, raising questions about whether psychosocial care after CBRN events should differ from that of non‐CBRN disasters [28]. The Bhopal incident in India highlighted the urgent need for mental health services for traumatized people and revealed the lack of support networks [11, 29, 30].

Addressing the traumatic effects of CBRN terrorism requires interventions at the societal level [29]. The findings of different studies indicate the reduction of the symptoms of anxiety, depression, and PTSD, as well as the improvement of mood ratings, communication and safety experience by psychological support and psychological first aid (PFA) [31, 32]. PFA is a psychosocial support activity designed to help people affected by emergencies, disasters, or traumatic events. PFA can be implemented in a safe environment and is based on five key principles: fostering hope, self‐efficacy, collective efficacy, social connectedness, safety, and relaxation [33]. The main goal of psychosocial support after CBRN incidents is to strengthen the connection between families and communities, eliminate chaos and uncertainty, implement psychosocial interventions, prevent psychological contradictions, rebuild and strengthen social and family ties, empower victims to recognize their capacities and flexibility, and support the journey to normality as a result of increasing coping mechanisms and the recovery process with consideration of vulnerable groups [34]. Pregnant women and infants are especially exposed to the adverse effects of social disasters [28]. Pregnancy involves a complex interplay of physiological, hormonal, and emotional factors that contribute to heightened emotional responses [19, 20, 21]. Recent events, such as pandemics, have changed women's perceptions and led to increased feelings of risk and anxiety, which can have unintended consequences on birth experiences and maternal and child distress [16, 35]. Concerns after a CBRN incident are significant for pregnant women and mothers with young children, some of whom consider abortion to prevent possible birth defects, requiring specialist training and counseling [36].

In this regard, the result of a systematic review emphasized the importance of risk communication, education, and psychosocial counseling for pregnant women, but noted a lack of strong evidence on their effectiveness [28]. Another systematic review study conducted on psychological interventions for CBRN incidents have not specifically addressed pregnant women [28]. In line with this, Abera et al. [37] conducted a systematic review and meta‐analysis demonstrating that relaxation‐based interventions in pregnant women significantly improved both maternal psychological well‐being and neonatal health outcomes. However, their review focused primarily on general pregnancy‐related stress, without addressing disaster‐related or CBRN‐specific psychological contexts. This gap further supports the need for the current review, which focuses specifically on psychological interventions for pregnant women exposed to CBRN events [37].

Therefore, most guidelines are general and do not address the specific needs of this vulnerable population. Many recommendations are based on expert opinion or unsystematic reviews, limiting evidence‐based decision‐making [38, 39, 40]. Therefore, Conducting a systematic review is essential to consolidating research, identifying gaps, improving mental health outcomes, and guiding policies and training for healthcare providers. This study examines psychological interventions for pregnant women in CBRN events, assessing their effectiveness and highlighting future research needs.

## 2 Methods

### 2.1 Study Design

This systematic review was conducted in accordance with the Preferred Reporting Items for Systematic Reviews and Meta‐Analyses (PRISMA) guidelines [41]. The study protocol was prospectively registered with PROSPERO (registration number: CRD4201082451).

### 2.2 Eligibility Criteria

Quantitative and qualitative studies published in English, without time limit, focusing on psychological interventions before, during, or after CBRN incidents for pregnant women affected by CBRN incidents, were included. Conference abstracts, non‐English studies, and studies without full‐text availability were excluded.

### 2.3 Selection Process

Two authors independently performed a systematic and comprehensive search of the PubMed, Embase, Web of Science, and Scopus databases up to November 4, 2023, with an updated search conducted in July 2025, which identified no additional eligible studies. An expert searcher was consulted during the development of the search strategy, and tailored strategies were designed for each database. The strategy was constructed using a combination of Medical Subject Headings (MeSH) terms and general keyword terms (Table 1).

**Table 1:** Final search strings and parameters used in the systematic search process.

Database	Search feilds	(Search string)
PubMed	Title/Abstract [tiab]	#1 (pregnant woman[tiab] OR pregnant women[tiab] OR pregnanc*[tiab] OR gestation[tiab])
		#2 (vulnerable population*[tiab] OR underserved population*[tiab] OR disadvantaged population*[tiab] OR sensitive population*[tiab])
		#3 (psychosocial intervention*[tiab] OR psychological intervention*[tiab] OR psychotherap*[tiab] OR psychological[tiab] OR psychosocial[tiab])
		#4 (CBRNE[tiab] OR CBRN[tiab] OR “CBRNE exposure*”[tiab] OR “CBRNE event*”[tiab] OR chemical[tiab] OR biological[tiab] OR radiological[tiab] OR nuclear[tiab] OR explosive[tiab])
		Final: #1 AND #2 AND #3 AND #4
Embase	Title, Abstract [ti,ab]	#1 (‘pregnant woman’:ti,ab OR ‘pregnant women’:ti,ab OR pregnanc*:ti,ab OR gestation:ti,ab)
		#2 (‘vulnerable population*’:ti,ab OR ‘underserved population*’:ti,ab OR ‘disadvantaged population*’:ti,ab OR 'sensitive population*’:ti,ab)
		#3 (psychosocial intervention*:ti,ab OR psychological intervention*:ti,ab OR psychotherap*:ti,ab OR psychological:ti,ab OR psychosocial:ti,ab)
		#4 (CBRNE:ti,ab OR CBRN:ti,ab OR ‘CBRNE exposure*’:ti,ab OR ‘CBRNE event*’:ti,ab OR chemical:ti,ab OR biological:ti,ab OR radiological:ti,ab OR nuclear:ti,ab OR explosive:ti,ab)
		Final: #1 AND #2 AND #3 AND #4
Scopus	Title, Abstract, Keywords [TITLE‐ABS‐KEY]	#1 (“pregnant woman” OR “pregnant women” OR pregnanc* OR gestation)
		#2 (“vulnerable population*” OR “underserved population*” OR “disadvantaged population*” OR “sensitive population*”)
		#3 (“psychosocial intervention*” OR “psychological intervention*” OR psychotherap* OR psychological OR psychosocial)
		#4 (CBRNE OR CBRN OR “CBRNE exposure*” OR “CBRNE event*” OR chemical OR biological OR radiological OR nuclear OR explosive)
		Final: #1 AND #2 AND #3 AND #4
Web of Science	Topic [TS]	#1 (“pregnant woman” OR “pregnant women” OR pregnanc* OR gestation)
		#2 (“vulnerable population*” OR “underserved population*” OR “disadvantaged population*” OR “sensitive population*”)
		#3 (“psychosocial intervention*” OR “psychological intervention*” OR psychotherap* OR psychological OR psychosocial)
		#4 (CBRNE OR CBRN OR “CBRNE exposure*” OR “CBRNE event*” OR chemical OR biological OR radiological OR nuclear OR explosive)
		Final: #1 AND #2 AND #3 AND #4

Search results were imported into EndNote, where duplicate studies were automatically removed. Two authors independently screened the titles, abstracts, and full texts based on the inclusion criteria. Any discrepancies were resolved through discussion, and if needed, by involving a third reviewer. Although formal inter‐rater reliability statistics were not calculated, the process ensured consistent and rigorous screening.

### 2.4 Data Collection Process

After screening the full texts, relevant data—including author(s), article title, publication year, country, study design, and type of intervention—were extracted from the eligible studies. The first author conducted an initial review of all titles, abstracts, and full texts. If uncertainty arose regarding an abstract's eligibility, it was further assessed by the second and third researchers. This approach ensured that 20% of the abstracts underwent additional review. In cases where ambiguity persisted, all three investigators discussed the abstract to reach a consensus on inclusion or exclusion. Any disagreements were resolved through discussion.

Inclusion criteria required that studies [1] involved pregnant women exposed to CBRN incidents [2]; evaluated psychological interventions aimed at reducing mental health symptoms or improving psychological well‐being; and [3] were research articles published in English. Exclusion criteria included studies that lacked a psychological intervention focus, were not related to CBRN exposure, or were commentaries or protocols. Psychological interventions were operationally defined as any structured mental health strategy such as cognitive behavioral therapy, psychoeducation, relaxation techniques, or PFA.

The selected studies specifically focused on psychological interventions related to pregnant women and CBRN events. Following multiple iterations of search queries utilizing various Boolean operators, a final set of studies was identified. The extracted data were then coded using MAXQDA Pro 2020, aligning with the research question. During thematic analysis, codes, subcategories, and concise categories were systematically identified. When similar concepts appeared within a shared context, they were integrated to establish broader categories.

### 2.5 Risk of Bias and Quality Assessment

The reporting quality and risk of bias were assessed separately. The methodological quality and risk of bias of the included studies were independently appraised by two reviewers using established checklists appropriate to each study design. Reporting quality of observational studies (cohort, case‐control, and cross‐sectional) were assessed using the STROBE statement, a validated 22‐item checklist focusing on transparent and comprehensive reporting [42]. Randomized controlled trials (RCTs) were evaluated with the CONSORT checklist, which addresses key aspects of trial design, conduct, analysis, and reporting to minimize bias [43]. Qualitative studies were appraised via the COREQ checklist, emphasizing rigor in data collection, analysis, and researcher reflexivity [44]. For mixed‐method studies, Mixed Methods Appraisal Tool (MMAT) was applied to ensure thorough quality assessment across quantitative and qualitative components [45]. Any discrepancies in scoring were reconciled through consensus discussion. This systematic quality appraisal enabled identification of potential biases, ensuring a robust synthesis of evidence within this review.

To assess the risk of bias in the included studies, appropriate tools were selected based on the study design. The Newcastle–Ottawa Scale (NOS) was applied to evaluate the methodological quality of observational studies [46]. For qualitative research, the Consolidated Criteria for Reporting Qualitative Research (COREQ) checklist was used [47]. RCTs were assessed using the revised Cochrane risk‐of‐bias tool (RoB 2) [48], while quasi‐experimental studies were evaluated using the ROBINS‐I tool [49]. Systematic reviews were appraised using the AMSTAR‐2 checklist [50]. Narrative reviews, due to the lack of systematic methodology, were not formally assessed for risk of bias. All assessments were independently performed by two reviewers, and disagreements were resolved through discussion or consultation with a third reviewer.

### 2.6 Data Extraction and Analysis

For each included study, data were extracted regarding study design, population characteristics, intervention details, outcome measures, and key findings. Quantitative and qualitative studies were handled separately during analysis. Quantitative studies were summarized descriptively. For qualitative studies, a thematic analysis was conducted to identify recurring concepts, patterns, and themes related to psychological support for pregnant women in CBRN contexts.A meta‐analysis was initially considered as part of the review process. However, following the full‐text screening and data extraction, it was determined that conducting a quantitative synthesis was not feasible. Only a small number of included studies were RCTs, and these showed substantial heterogeneity in their design, population, intervention types, outcome measures, and reporting formats. In addition, several RCTs lacked the statistical details necessary for inclusion in a pooled analysis (e.g., means, standard deviations, or effect sizes). As a result, a narrative synthesis was undertaken. Studies were grouped and compared based on methodological design, type of psychological intervention, and the main outcomes reported. This approach allowed for a descriptive comparison of the evidence without introducing the potential biases associated with inappropriate data pooling. Finally, the findings from both qualitative and quantitative analyses were integrated narratively in the discussion section to provide a comprehensive understanding of psychological interventions for pregnant women exposed to CBRN events.

## 3 Results

### 3.1 Selection

**Figure 1:**
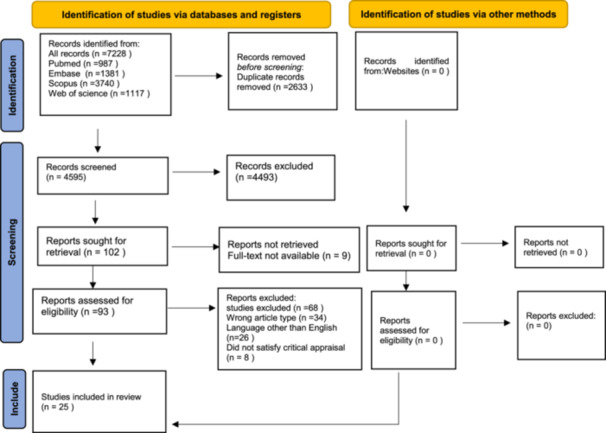
PRISMA flow diagram showing the study selection process.

The systematic searches across the databases returned 7228 records, including 987 studies from Pubmed, 3740 from Scopus, 1117 from Web of science, and a manual search from Embase yielded 1381 reports (see Figure 1). After removing the duplicates, 4595 records were screened based on title and abstract. This phase removed titles/abstracts that did not meet the eligibility criteria (4493). Thus, a full‐text analysis has been done for the remaining 102 studies. Subsequently, 77 reports were eliminated due to various reasons, such as 34 Wrong article type, 26 Language other than English, 9 Conference abstract only, 8 Did not satisfy critical appraisal, 25 studies remained within the purview of analysis (Table 2).

**Table 2:** Review of 25 Mamel articles in terms of repetition in the study (full article).

Study	Frequency
Cross‐sectional survey	11
Review and systematic review and meta‐analysis	6
Quantitative descriptive	1
Prospective	1
Evidence‐based interventions	1
Mixed‐methods study	1
Qualitative analysis	1
Randomized clinical tria	2
Quasi experiment	1

### 3.2 Risk of Bias and Quality Assessment

Out of 25 included studies, 19 were eligible for formal quality assessment using validated tools tailored to each study design. Overall, the majority of studies (*n* = 13) were rated as good quality, while 6 studies received a moderate rating. Cross‐sectional studies assessed with the STROBE checklist generally demonstrated acceptable reporting quality, with scores ranging from 67% to 92%. RCTs evaluated via the CONSORT checklist showed high methodological quality (≥ 80%). Mixed‐method, quasi‐experimental, and systematic review designs also met most quality criteria using MMAT, TREND, and AMSTAR‐2 tools, respectively. Narrative and non‐systematic reviews (*n* = 6) were excluded from quality scoring due to lack of standardized evaluation frameworks.

Furthermore, out of the 25 included studies, six were assessed as having a low risk of bias, primarily including RCTs and systematic or rapid reviews with clear methodologies and complete reporting (e.g., Zendehdel et al. [51], Güney et al. [52], Gouweloos et al. [28], Ahmad & Vismara [53]). The majority of studies (*n* = 19) demonstrated a moderate risk of bias, often due to limitations such as self‐reported data, non‐random sampling, or lack of systematic synthesis, especially among cross‐sectional and narrative designs. Only one study showed low risk in a prospective cohort format (Levi et al. [54]). No study was assessed as having a high risk of bias. Narrative and descriptive reviews were included for contextual insights but were not subject to formal bias appraisal tools (Supporting Information S1: Appendix S1).

### 3.3 Study Characteristics

The research showed that most of the studies were conducted in Japan (*n* = 8) [19, 21, 34, 35, 38, 55, 56, 57], while three studies were conducted in Iran [20, 51, 58], two studies were conducted in Canada [59, 60], 2 studies were conducted in Turkey [52, 61], and other studies were conducted in the United States of America [16], the Netherlands [28], Jimma Ethiopia [37], West Germany [62], Romania [63], China [64], Sweden [54], Italy [65], Qatar [66], and Indonesia [67]. These studies were published between 1983 and 2023. The total sample size excluding systematic reviews and review studies among the studies was 124,430. Most of the articles are related to post‐traumatic events, with the longest mean follow‐up time from 2018 to 2011. The data of the entered studies including the title, author's name, year of publication, sample size, type of study and psychological interventions were extracted. A summary of key data from the included studies is presented in Table 3.

**Table 3:** Categorized findings of psychological support measures for pregnant women based on reviewed literature.

	Author	Country/year	Design	Sample	Psychological interventions designed for pregnant women.
1	Aya Goto et al. [35]	Apan/2017	Cross‐sectional surveys	13,109	− Telephone counseling − Maternal mental health support − Child health check‐ups − Enhancing social support
2	Ito, Shinya et al. [19]	Apan/2017	Qualitative analysis	12,415	− Information on radiation risks − Long‐term maternal mental health support − Parental education and empowerment
3	Marzieh Masjoudi, et al. [20]	Iran/2020	A mixed‐methods study with a sequential explanatory design	215	− Online care services − Psychological counseling and social support − Education on COVID‐19, self‐care, vaccination
4	Juul Gouweloos et al. [28]	Netherlands/2014	A systematic literature review	—	− Risk communication − Psychosocial support programs − Community connection and security planning
5	Akira Ohtsuru, Koichi et al. [21]	Japan/2015	The article and evidence‐based interventions	—	− Mental health services for refugees − Coordination between organizations − Awareness and continuity of care
6	Sena D. Aksoy et al. [61]	Turkey/between March 2021 and March 2022	A quantitative descriptive study	326	− Increased social support − Healthcare access − Positive health behaviors
7	Louise Lemyre [59]	Canada/2005	Review	—	− Risk communication − Social support and education − Psychosocial risk management
8	Monica Ahmad and Laura Vismara [53]	December 2020 to January 2021	Review	—	− Physical activity − Social support − Emotional expression through digital tools
9	Shaoqi Chen [64]	China/2020	Cross‐sectional study	1160	− Self‐entertainment activities − Communication with others − Online consultations
10	Ruxandra‐Gabriela Cigăran et al. [63]	Romania/May and October 2020	Cross‐sectional survey	557	− Mental health support − Psychological support during maternity care − Multidisciplinary approach
11	Masaharu Maeda, Misari Oe, and Yuriko Suzuki [34]	Japan/2018	Review	—	− Screening tools for psychiatric risk − Public anti‐stigma campaigns − Financial support for care network
12	Ragnar Levi et al. [54]	Sweden/1989	Prospective study	86	− Support from maternity healthcare − Social support − Midwifery‐psychological interaction
13	Aya Goto et al. [55]	Japan/2010–2011	Prefectural wide cross sectional study	8196	− Mental health prioritization − Strategic parent support − Maternity care continuity
14	Kayoko Ishii et al. [68]	Japan/2021	A survey	24,444	− Telephone counseling − Radiation anxiety mitigation − Routine care for pregnant/lactating mothers
15	Claudia Ravaldi et al. [65]	Italy/2019	Cross sectional	(200)	− Respectful alliance with women − Attention to psychological history − Empowerment through support programs
16	Leili Salehi et al. [58]	Iran/2020	Cross‐sectional	222	− COVID‐19 awareness education − Managing anxiety from care access issues − Pregnancy joy promotion during pandemic
17	Tom Farrell et al. [66]	Qatar/2020	Cross‐sectional survey	288	− Coping strategy education − Support from maternity staff − Public health education
18	Catherine Lebel et al. [60]	Canada/2020	Survey	1987	− Social support − Physical activity − Anxiety/stress reduction planning
19	Masaharu Maeda et al. [56]	Japan	Review	—	− Annual telephone counseling − Interdisciplinary mental health team − Suicide and stigma prevention
20	Kayoko Ishii et al. [69]	Japan/2011 to 2014/2017	Survey	60,860	− Parenting support − Long‐term mental healthcare − Counseling and stigma reduction
21	Shinya Ito et al. [70]	Japan/2018	Survey	310	− Radiation health education − Long‐term support for anxious mothers − Anti‐stigma support
22	Juli Gladis Claudia et al. [67]	Indonesia/2020	Quasi experiment	60	− Lemon aromatherapy − Health education
23	Pooja Nadholta et al. [71]	2020	Literature review	—	− Stress reduction through yoga − Routine pregnancy care
24	Mojgan Zendehdel et al. [51]	Iran/2020	Randomized clinical trial	126	− Progressive muscle relaxation − Exercise programs
25	Esra Güney et al. [52]	Turkey/2020	Randomized clinical trial	84	− Online Mindfulness‐Based Stress Reduction (MBSR) program − Meditation techniques − Psychological support

*Note:* Full descriptions of the psychological interventions for each study are provided in Supporting Information S2: Appendix S2.

Pregnant women are usually vulnerable when accidents happen and need extra support. This vulnerable group requires special attention from physicians, midwives, public health nurses, and psychotherapists, including case identification, triaged care, and the provision of appropriate mental health interventions integrated with emergency medical responses and trauma care [72].

**Figure 2:**
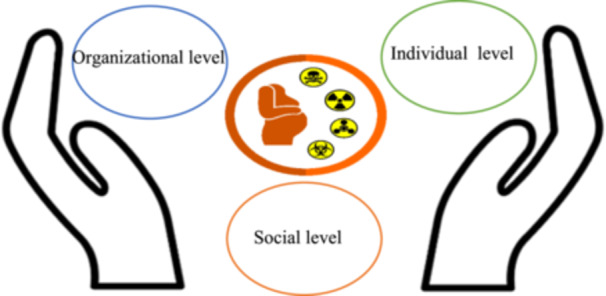
Outline of psychological interventions on pregnant women in CBRN incidents.

In this study, we examined the types of these interventions in pregnant women and based on the findings, the interventions can be classified into three categories: individual, organizational, and social (Figure 2). This thematic classification provides a conceptual foundation; however, a critical comparison of interventions—based on effectiveness, scalability, and context—was also incorporated to ensure analytical depth (Table S4 in Supporting Information S3: Appendix S3‐a). Key themes are summarized in the main text, while detailed study‐level findings and supporting evidence are presented in Supporting Information S3: Appendix S3‐b.

#### 3.3.1 Individual Level

Several studies have highlighted the role of physical self‐care in promoting psychological resilience among pregnant women during CBRN and pandemic events. Increased physical activity and engagement in informal self‐care strategies were associated with reduced psychological symptoms, although most evidence relied on self‐reported outcomes and cross‐sectional designs [53, 54, 60, 64]. Overall, physical self‐care appears to be a promising supportive approach, but more structured and evidence‐based interventions are needed in crisis settings.

##### 3.3.1.1 Psychological Health‐Promoting Behaviors

Relaxation‐based and mindfulness interventions, including progressive muscle relaxation, yoga, aromatherapy, and meditation, have been shown to reduce stress and anxiety and improve overall well‐being among pregnant women during pandemic and crisis situations [50, 52, 58, 67, 71]. While these interventions appear promising, the evidence is limited by small sample sizes, short‐term follow‐up, and reliance on self‐reported outcomes.

#### 3.3.2 Organizational Level

Organizational‐level interventions emphasize strengthening healthcare systems, ensuring continuity of maternal services, and integrating psychosocial support within disaster and pandemic response frameworks. The second main category was the “organizational level,”, derived from four subcategories: “Psychosocial and Maternal Health Support Infrastructure and Education,” “Maternal and Child Healthcare services,” “Mental Health Support for Mothers,” “Disaster response and Risk Communication.”

##### 3.3.2.1 Acsess to Suppport System and Education

Access to support systems and education at the organizational level is essential for protecting maternal mental health during CCBRN and pandemic‐related crises. Studies emphasize the importance of psychosocial support infrastructure, including ongoing regional monitoring, parenting support, and counseling services to ensure continuity of care, particularly in evacuation or disrupted healthcare settings [21, 34, 69].

Organizational approaches also highlight the value of involving pregnant women in healthcare decision‐making, which may enhance well‐being and autonomy, although evidence is often limited by cross‐sectional designs [18]. Multidisciplinary obstetric care and integrated psychological support have been repeatedly identified as necessary, yet challenges remain in implementing such models consistently across settings [63].

Furthermore, coordinated collaboration among healthcare professionals, government, and community organizations, alongside strengthened screening tools and sustainable care networks, has been recommended to improve preparedness and long‐term support during crises [19, 25, 31, 50]. Overall, these findings suggest that structured organizational systems and social support networks are critical components of maternal psychosocial care in emergency contexts [20, 21, 28, 35, 53, 60].

##### 3.3.2.2 Maternal and Child Healthcare Services

Maintaining continuity of prenatal and postnatal care, including birth planning support, postpartum services, vaccination, and parental guidance, is a critical organizational priority during disasters and pandemics [18]. In addition, integrating psychological and developmental health examinations for children has been recommended to indirectly support maternal well‐being, although standardized protocols remain limited [35, 69].

##### 3.3.2.3 Mental Health Support for Mothers

The literature highlights the importance of accessible maternal mental health support during disasters and pandemics, including counseling, midwife‐ and nurse‐led interventions, and long‐term psychosocial follow‐up [35, 55, 70]. Higher levels of depression and anxiety have been associated with crisis‐related concerns, disrupted prenatal care, relationship strain, and social isolation, underscoring the need for sustained and scalable support models [20, 60].

##### 3.3.2.4 Disaster Response and Risk Communication

Effective disaster response and risk communication interventions aim to reduce anxiety by providing accurate information, countering misinformation, and strengthening public trust [19, 20, 28, 34, 54, 58, 59, 61, 66, 70, 73, 74]. Key strategies include regular monitoring of maternal health, assessing information needs, providing detailed guidance about symptoms and risks, promoting parent and responder training, encouraging physical activity, and utilizing tele‐psychology for remote support. Overall, proactive communication and supportive interventions are essential to mitigate psychological impacts on pregnant women during CBRN and pandemic emergencies, though further research is needed to confirm long‐term effectiveness [19, 20, 28, 34, 60, 74].

#### 3.3.3 Social Level

Social‐level interventions focus on community‐based support networks, public initiatives, and long‐term coordination to enhance resilience among pregnant women during crises [20, 21, 28, 34, 35, 58, 59, 60, 61, 65, 68]. Key strategies include providing emotional, informational, and practical support through families, neighbors, local organizations, and institutions; implementing public anti‐stigma campaigns; ensuring continuity of maternal healthcare; planning evacuations for vulnerable populations; and facilitating remote or community‐based health services. Overall, community initiatives and social support networks are essential to reduce maternal distress and enhance coping during disasters, though their effectiveness may vary depending on context and implementation [20, 21, 28, 34, 58, 65, 68].

##### 3.3.3.1 Long‐Term Planning and Coordination

Long‐term planning and coordination highlight the need for sustained psychosocial support, continuity of care, and effective resource allocation for mothers and families affected by CBRN‐related crises. Core components include long‐term mental healthcare networks, stigma reduction initiatives, and ongoing risk communication to address psychological distress and social consequences. Overall, coordinated and sustained planning is essential, although empirical evidence on the long‐term effectiveness of these interventions remains limited [19, 28, 56].

## 4 Discussion

This systematic review examines psychological interventions for pregnant women during CBRN incidents based on 25 studies. Notably, no study has addressed psychological interventions for pregnant women in chemical incidents. Among the 25 studies, 10 focus specifically on interventions in nuclear and radiological incidents, 13 examine interventions during biological incidents (such as pandemics and infectious diseases), and 2 studies address general CBRN events.

This imbalance in the distribution of studies—where most research focuses on biological (e.g., pandemics) and nuclear/radiological incidents, with minimal attention to chemical events—likely reflects global health priorities shaped by high‐impact emergencies such as the H1N1 influenza outbreak, the COVID‐19 pandemic, and the Fukushima nuclear disaster [75, 76, 77]. These events triggered major policy and research investments, leading to a disproportionate body of evidence in those areas. However, the lack of studies on psychological interventions in chemical CBRN incidents is concerning, particularly given the high risk of exposure to toxic agents in industrial or terrorist contexts. This research gap may result in inadequate preparedness and support for vulnerable populations, such as pregnant women, in chemical emergencies. Addressing this imbalance is crucial to ensuring more equitable and comprehensive health system readiness across all CBRN threats.

Interventions identified in this study were classified into three levels: individual, organizational, and social. Given the pandemic's impact on personal autonomy, it is essential to consider women's active participation in decisions related to self‐care. In the present research, physical self‐care and psychological health‐promoting behaviors were identified as subcategories of individual level. Monteith and Pearce's study on self‐care decontamination in case of chemical exposure highlighted the importance of self‐care in CBRN incidents [78]. Furthermore, Gouweloos et al. [28], in a review study about psychosocial care to affected citizens and communities in CBRN incidents, showed that individuals should receive psychosocial counseling and care after CBRN attacks. Citizens can also improve their psychological well‐being by adopting coping strategies such as accepting reality, building support networks, and managing stress. The study also emphasizes that providing accurate and transparent information about risks and preventive measures to citizens helps reduce anxiety and enhances their ability to cope with crises. Pregnant women should also receive specialized psychological care to reduce the risk of miscarriage or other negative impacts on the unborn child [28]. Nevertheless, these findings were often generalized, with limited evidence about long‐term outcomes or adaptation in culturally diverse settings. Some studies even lacked detailed implementation protocols, making it difficult to assess effectiveness in real‐world emergencies.

Cultural considerations also play a key role. Farrell et al. showed that cultural beliefs in the Middle East region traditionally perceive exercising during pregnancy as dangerous, which contributes to low physical activity in this population [66].

This points to the importance of integrating cultural sensitivity in intervention design, ensuring that proposed self‐care practices are both culturally acceptable and evidence‐based.

The present research highlighted another category entitled “Organizational level.” It emerged from four subcategories included: “Psychosocial and Maternal Health Support Infrastructure and Education”, “Maternal and Child Healthcare services”, “Mental Health Support for Mothers”, “Disaster response and Risk Communication.”

A study by Hamachandra was conducted in Sri Lanka due to the increasing nature of disaster risks and the status of women's empowerment in risk governance structures. Twenty challenges were identified and categorized into five broad themes: legal, institutional, individual, social and cultural, and the nature of the job. The study emphasized the importance of psychological support and also highlighted the lack of gender integration in the national strategic plan. This included areas such as political and civil rights, family rights, access to education and training, economic rights and benefits, healthcare and nutrition, protection from social discrimination, and safeguarding against gender‐based violence [79]. These structural and systemic gaps—particularly in gender‐specific emergency planning—underscore why psychological interventions must not only focus on individual behavior but also address institutional readiness and inclusivity.

In the article “Recommended Psychological Crisis Intervention Response to the 2019 Novel Coronavirus Outbreak in China,” it was emphasized that the pandemic caused severe psychological effects, particularly among those in quarantine. The psychological intervention model provided by West China Hospital utilized internet technology to connect doctors, psychiatrists, psychologists, and social workers, offering psychological care to patients, their families, and medical staff [23]. This model shows how digital tools can support mental health in future CBRN crises.

Also, the study of Ravaldi et al. conducted on pregnant women during the Corona period showed that women think that this disease minimizes the feeling of empowerment and threatens their well‐being and health in the short and long term. Midwives and obstetricians can help women regain their potential, imagination and confidence in themselves and their caregivers by considering women's feelings and their specific needs and creating a respectful alliance and empowering women. With this in mind, ensuring compliance with WHO rules can reassure women about their basic rights and enable them to achieve the best possible birth experience with current restrictions [65]. These findings highlight the need to support pregnant women with respectful care, mental health services, and standard‐based childbirth during CBRNe crises.

During CBRNe events, policies must counter misinformation through evidence‐based communication tailored to vulnerable populations such as pregnant women. Effective risk communication strategies—especially in culturally diverse contexts—are essential for mitigating psychological distress. Monteith et al. and Soltani et al. highlighted the need for accurate and scientific communication about radiation risks to pregnant women to reduce anxiety [78, 80]. Similarly, Ravaldi et al. [65] noted that pregnant women rely heavily on the internet and healthcare professionals for trustworthy COVID‐19 pregnancy information. Hemachandra et al. [79] emphasized that organizations must develop culturally appropriate mental health support plans and counter misinformation during CBRNe events through effective evidence‐based communication, especially targeting vulnerable populations. Past disasters like Chernobyl and COVID‐19 revealed how unclear information hindered responses and undermined resilience [57, 76, 77]. For policymakers, this highlighted the urgent need to integrate clear, consistent, and culturally competent risk communication into emergency preparedness plans.

Remote psychological support and AI tools offer free and accessible care, as evidenced by Fukushima's survey highlighting maternal health assessments and telephone counseling [40]. PFA, presented by Gillespie [81, 82], is a critical psychosocial intervention to mitigate trauma effects after disasters, though Brown et al. [83] caution that survivors' complex reactions may delay healing. Social support is crucial for mental and physical health, promoting positive emotions, regulation, and stress reduction [84, 85, 86]. Remote support, PFA, and social care should be core components of CBRNe response for pregnant women.

Fukushima Medical University's pregnancy survey underscored parental support, long‐term mental healthcare, stigma reduction, and maternal‐child health resources as key needs [69]. The MorgansteinJC_PandemHealthCareEmergenc study states that community dynamics should be strengthened and restored using structured and spontaneous psychosocial support approaches and assign roles for collective participation that increase resources, reduce feelings of powerlessness, and sense of belonging. Fosters hope. Also, ongoing mental health surveillance should be implemented to guide the allocation of services and financial support to those most at risk of PTSD, depression, substance dependence, and broader psychosocial challenges [87].

These studies often miss detailed evaluation of interventions for pregnant women in CBRN settings, creating a gap in tailored strategies. More research is needed to develop interventions addressing their unique psychosocial and medical needs.

Overall, the studies emphasize the critical need for comprehensive psychosocial support for pregnant women in CBRN incidents, as the psychological effects can be severe and long‐lasting. Effective interventions are necessary to reduce adverse effects and promote positive outcomes for both women and their children. This includes strengthening individual health, providing appropriate health and medical services, creating social support, and providing the necessary financial and legal resources.

Finally, Social level category in the present study had two subcategories included “community initiatives” and “Long‐term Planning and Coordination.” Mani and et al. [88] in their study about Public Health Responses to CBRN Terrorism in Africa highlighted the importance of enhancing healthcare preparedness, strengthening emergency response systems, and establishing sustainable public health policies. Also, their findings revealed extensive effects of CBRN terrorism on healthcare systems, emphasizing the difficulties in delivering immediate medical care, community and the critical need for comprehensive strategies to support long‐term recovery efforts and coordination. This study showed nessissity of public education, recognizing early signs of exposure, psychological support, international collaborations. This findings were similar to our study. However, the present study emphesised on pregnant women in CBRN incidents [88]. These insights align with our findings but place special emphasis on pregnant women in CBRN contexts.

Providing telehealth resources, including providing trusted medical apps and/or websites that women can use to increase their understanding of pregnancy. Collaborate with physicians when making decisions about policies and procedures and ensure that their opinions and expertise are included and respected. Increasing access to support personnel such as social workers and encouraging their collaboration with perinatal physicians to help nurses and midwives adapt to women in challenging situations [64]. The study by Ruxandra‐Gabriela Cigăran et al. [63] showed that the main concern of pregnant women is related to threats to the life and health of their baby due to the uncertainty caused by the covid disease. Researchers emphasized knowing the negative effects of maternity care changes and restrictions and suggested the need for psychological support to improve their mental health and prevent negative pregnancy outcomes [63]. Specific guidance for pregnant women is not readily available. This absence of guidelines is especially notable for exposures associated with emergency and existing exposure situations. Furthermore, the biokinetic specificities of individual radionuclides have to be taken into account [89].

### 4.1 Limitations of the Study

The present review was limited to articles published in English, which may have introduced selection bias. Furthermore, publication bias cannot be ruled out, and the lack of quantitative synthesis reflects the heterogeneity of included studies. While meta‐analysis is a robust method for integrating findings across studies, the included articles exhibited considerable heterogeneity in terms of design, interventions, and outcome reporting. Moreover, most studies lacked the statistical data required for quantitative synthesis. Therefore, performing a meta‐analysis was deemed methodologically inappropriate.

## 5 Conclusion

In summary, the complex picture of psychosocial consequences resulting from CBRN incidents requires a nuanced and comprehensive approach to intervention. This study has investigated effective psychological interventions to reduce the psychological impact of these incidents on the population of pregnant women and how to support them in their journey towards recovery and normalcy. These interventions are applicable at three individual, organizational and social levels. It should be noted that the joint efforts of healthcare providers, policymakers, and community organizations are critical in this effort and ensure that psychosocial care provided is comprehensive, accessible, and sensitive to the distinct needs of affected individuals. Finally, the goal is not only to reduce the immediate distress, but also to empower individuals and communities to rebuild and flourish after such devastating events and prevent psychological consequences for children in the future.

## Author Contributions

M.A. and Z.C. designed the search strategy, while Z.C. conducted the literature search. M.A., Z.C., S.S., F.T., and M.S. performed study selection, data extraction, and quality assurance. M.S. carried out the qualitative data analysis. M.A. and Z.C. prepared the initial draft of the manuscript, and all authors reviewed and approved the final version. M.A. and M.S. supervised the project, with Maryam Azizi, the corresponding author, having full access to all data and taking responsibility for the integrity and accuracy of the data analysis. All authors contributed to the conceptualization of the systematic search.

## Funding

The authors have nothing to report.

## Ethics Statement

The authors have nothing to report.

## Consent

The authors have nothing to report.

## Conflicts of Interest

The authors declare no conflicts of interest.

## Transparency Statement

The lead author, Maryam Azizi, affirms that this manuscript is an honest, accurate, and transparent account of the study being reported; that no important aspects of the study have been omitted; and that any discrepancies from the study as planned (and, if relevant, registered) have been explained.

## Supporting information

Supporting File 1

Supporting File 2

Supporting File 3

## Data Availability

The data that supports the findings of this study are available in the Supporting Information [Link], [Link], [Link] of this article.
