# Supplementary material for: Psychological Interventions for Pregnant Women in Chemical, Biological, Radiological, and Nuclear Incidents: A Systematic Review
Source: Health Sci Rep. 2026 Jun 2;9(6):e72217. doi: 10.1002/hsr2.72217 (PMC13239930; doi:10.1002/hsr2.72217)
Supplement: Supplementary file 1 — Supporting File 1 [file HSR2-9-e72217-s002.docx]

| **No.** | **Author, Year** | **Study Type** | **Checklist Used** | **Total Items** | **Items Met** | **Quality Percentage** | **Quality Rating** |
| --- | --- | --- | --- | --- | --- | --- | --- |
| 1 | Aya Goto et al, 2017 | Cross-sectional | STROBE | 12 | 10 | 83% | Good |
| 2 | Ito et al, 2017 | Qualitative | COREQ | 15 | 11 | 73% | Moderate |
| 3 | Masjoudi et al, 2020 | Mixed-methods | MMAT | 15 | 13 | 87% | Good |
| 4 | Juul Gouweloos et al, 2014 | Systematic review | AMSTAR 2 | 16 | 14 | 88% | Good |
| 5 | Akira Ohtsuru et al, 2015 | Narrative review | - | - | - | - | - |
| 6 | Sena D. Aksoy et al, 2022 | Quantitative descriptive | STROBE | 12 | 9 | 75% | Moderate |
| 7 | L Lemyre, 2005 | Review | - | - | - | - | - |
| 8 | Ahmad & Vismara, 2020 | Rapid evidence review | - | - | - | - | - |
| 9 | Shaoqi Chen, 2020 | Cross-sectional | STROBE | 12 | 9 | 75% | Moderate |
| 10 | Ruxandra-Gabriela Cigăran et al | Cross-sectional survey | STROBE | 12 | 10 | 83% | Good |
| 11 | Maeda et al, 2018 | Review | - | - | - | - | - |
| 12 | R. Levi et al, 1989 | Prospective study | STROBE | 12 | 8 | 67% | Moderate |
| 13 | Aya Goto et al, 2010-2011 | Cross-sectional | STROBE | 12 | 11 | 92% | Good |
| 14 | Kayoko Ishii et al, 2011-2018 | Survey | STROBE | 12 | 10 | 83% | Good |
| 15 | Claudia Ravaldi et al, 2019 | Cross-sectional | STROBE | 12 | 9 | 75% | Moderate |
| 16 | L Salehi et al, 2020 | Cross-sectional | STROBE | 12 | 9 | 75% | Moderate |
| 17 | Tom Farrell et al, 2020 | Cross-sectional survey | STROBE | 12 | 8 | 67% | Moderate |
| 18 | Catherine Lebel et al, 2020 | Survey | STROBE | 12 | 10 | 83% | Good |
| 19 | Maeda et al, 2017 | Review | - | - | - | - | - |
| 20 | Kayoko Ishii et al, 2011-2017 | Survey | STROBE | 12 | 11 | 92% | Good |
| 21 | Shinya Ito et al, 2018 | Survey | STROBE | 12 | 10 | 83% | Good |
| 22 | Juli Gladis Claudia et al, 2020 | Quasi-experiment | TREND | 15 | 12 | 80% | Good |
| 23 | Pooja Nadholta et al, 2020 | Literature review | - | - | - | - | - |
| 24 | Mojgan Zendehdel et al, 2020 | RCT | CONSORT | 25 | 20 | 80% | Good |
| 25 | Esra Güney et al, 2020 | RCT | CONSORT | 25 | 22 | 88% | Good |

Quality ratings are based on the percentage of checklist items met: scores above 75% indicate **Good** quality, between 50% and 75% indicate **Moderate** quality, and below 50% indicate **Poor** quality.

| **No.** | **Study Title** | **Type of Study** | **Risk of Bias Tool** | **Summary of Risk of Bias** |
| --- | --- | --- | --- | --- |
| 1 | Goto et al. (2017) | Cross-sectional | NOS | Moderate risk: potential selection and self-reporting bias |
| 2 | Ito et al. (2017) | Qualitative | COREQ | Low risk: transparent methods and credible data |
| 3 | Masjoudi et al. (2020) | Mixed-method | MMAT | Moderate risk: sampling bias in quantitative component and limited integration of data strand |
| 4 | Gouweloos et al. (2014) | Systematic review | AMSTAR-2 | Low risk: complete reporting and clear methodology |
| 5 | Ohtsuru et al. (2015) | Narrative review | – | Moderate risk: lack of systematic search strategy |
| 6 | Aksoy et al. (2021) | Cross-sectional | NOS | Moderate risk: self-reported data and limited sampling |
| 7 | Lemyre (2005) | Narrative review | – | Moderate risk: unclear study selection and synthesis |
| 8 | Ahmad & Vismara (2020) | Rapid review | AMSTAR-2 | Low risk: clear reporting and validated methods |
| 9 | Chen (2020) | Cross-sectional | NOS | Moderate risk: sampling limitations and measurement tools |
| 10 | Cigăran et al. (2020) | Cross-sectional | NOS | Moderate risk: possible response and selection bias |
| 11 | Maeda et al. (2018) | Review | – | Moderate risk: general reporting, lacks systematic design |
| 12 | Levi et al. (1989) | Prospective cohort | NOS | Low risk: forward design and appropriate data collection |
| 13 | Goto et al. (2010–2011) | Cross-sectional | NOS | Moderate risk: potential sampling and response bias |
| 14 | Ishii et al. (2011–2018) | Survey | NOS | Moderate risk: limited measurement tools and follow-up |
| 15 | Ravaldi et al. (2019) | Cross-sectional | NOS | Moderate risk: risk of response bias |
| 16 | Salehi et al. (2020) | Cross-sectional | NOS | Moderate risk: self-reporting and sampling issues |
| 17 | Farrell et al. (2020) | Cross-sectional | NOS | Moderate risk: self-reported measures and sample selection |
| 18 | Lebel et al. (2020) | Cross-sectional | NOS | Moderate risk: possible response and sampling bias |
| 19 | Maeda et al. | Review | – | Moderate risk: narrative style with no systematic process |
| 20 | Ishii et al. (2011–2017) | Survey | NOS | Moderate risk: small and possibly biased sample |
| 21 | Ito et al. (2018) | Survey | NOS | Moderate risk: limited generalizability due to sample |
| 22 | Gladis et al. (2020) | Quasi-experimental | ROBINS-I | Moderate risk: lack of randomization and control over confounders |
| 23 | Nadholta et al. (2020) | Narrative review | – | Moderate risk: non-systematic reporting |
| 24 | Zendehdel et al. (2020) | Randomized Controlled Trial | RoB 2 | Low risk: standard RCT design and full reporting |
| 25 | Güney et al. (2020) | Randomized Controlled Trial | RoB 2 | Low risk: clear follow-up and comprehensive reporting |
