# Supplementary material for: Psychological Interventions for Pregnant Women in Chemical, Biological, Radiological, and Nuclear Incidents: A Systematic Review
Source: Health Sci Rep. 2026 Jun 2;9(6):e72217. doi: 10.1002/hsr2.72217 (PMC13239930; doi:10.1002/hsr2.72217)
Supplement: Supplementary file 2 — Supporting File 2 [file HSR2-9-e72217-s003.docx]

Appendix 3-a

Table 4: Thematic categorization of psychological interventions for pregnant women

| Categories | Sub-categories | Main codes |
| --- | --- | --- |
| Individual level | Physical Self-care | Exercise and physical activity  Following a proper nutritional diet  Effort to prevent getting sick  A change in routine activities  Self care practice  Educational programs on health risks |
|  | psychological health-promoting behaviors | Meditation  Relaxation techniques  Aromatherapy, yoga for stress reduction  Turning to simple hobbies  mindfulness  Increased resilience  Enjoying happiness caused by pregnancy experience  Comunication with family and others  Accepting problems  Addressing the unique needs of women with psychological histories Ensuring respectful care  Compatibility  Coping strategies |
| Organization level | Psychosocial and Maternal Health Support Infrastructure and Education | Access to health care and routine care  Referral and special treatment  Access to counseling and psychosocial support (online, telephone, professional)  Protection of pregnant workers and patients  Prioritizing pregnant women to receive care  Continuity of providing care  Multidisciplinary approach in providing care  Having respectful behavior with people  Involving pregnant women in decisions-making  Planning to secure government financial support such as insurance  Develop the current care network  Planning to foster good cooperation with other health professionals  Development of screening systems to identify individuals at risk for psychiatric disorders  Engaging social support networks for mothers, including family and friends  Coordinated efforts among healthcare professionals, government, and community organizations  Specialized training programs for pregnant women  General standards for psychosocial care after disasters  Financial support from the government  Policymakers aware of Providing guidance |
|  | Maternal and Child Healthcare services | Emphasis on prenatal and postnatal care  Child health examinations  Providing information about birth planning  Postpartum care  COVID-19 vaccination for pregnant and lactating women  Guide parents |
|  | Mental Health Support for Mothers | Telephone counseling and psychological support by midwives and nurses  Focus on maternal self-confidence  Addressing symptoms of maternal depression.  Long-term mental health support  Empowerment for mothers in post-disaster |
|  | Risk Communication | Attention to psychosocial care during CBRN incidents  Planning for effective risk communication to dispel misinformation and address stigma  Continuous assessment of mothers' health and addressing psychological factors like stress and anxiety  Rumor management  Management of disturbing information in the media  Assessment of information needs  Providing detailed information about the incident (extent of the incident, people at risk)  Development of risk communication skills  Providing detailed information about symptoms, diagnosis, treatment and complications  Public communication  Risk communication skills  Face-to-face, individual, group or virtual training  Staff training |
| Social level | Community Initiatives | Public Education such as evacuation  General and local social support  Implementing Public anti-stigma campaigns  Inter-regional and inter-community collaboration  Planning for public health services  Community support after disasters  Emphasis on the importance of reliable support programs and interregional cooperation.  Reduce anxiety and improve public mental health |
|  | Long-term Planning and Coordination | Planning for sustained support and resources for mothers and families affected by disasters.  Addressing stigma related to mental health  Addressing health risks associated with radiation exposure |

Appendix 3-b

Detailed Study-Level Findings of Interventions for Pregnant Women in CBRN

1. Individual level:

Interventions at the individual level are divided into two parts: Physical self-care and psychological health-promoting behaviors. The results of the studies that led to the formation of these subcategories are explained below in order:

- Physical self-care

Several studies have highlighted the role of physical self-care in promoting psychological resilience among pregnant women during CBRN and pandemic events. Ahmad and Vismara (50) as well as Lebel et al. (57) found that increased physical activity correlated with reduced psychological symptoms, positioning exercise as a potential protective factor. However, both studies lacked detail regarding the type and duration of activities, and their cross-sectional designs limited causal interpretation. Similarly, Chen (61) and Levi et al. (51) noted that women often engaged in informal self-care strategies—such as participating in health education programs, seeking social support, and searching for coping solutions online. While these actions appeared beneficial, the studies relied heavily on self-reports without standardized outcome measures. Moreover, the mechanisms of engaging women in such practices were not clearly described. These limitations underscore the need for structured, evidence-based self-care interventions tailored to crisis settings and supported by measurable psychological outcomes. Overall, physical self-care appears to be a promising supportive strategy, although more structured and evidence-based interventions are needed in crisis settings.

- psychological health-promoting behaviors

The results of Zendehdel at al’s study showed that progressive muscle relaxation is an effective intervention for reducing anxiety in pregnant women during the coronavirus pandemic and should be emphasized and sensitively recommended by healthcare providers as part of pregnancy care(47). This quasi-experimental study offered strong evidence for its intervention; however, long-term outcomes were not assessed.

Given its integrated approach combining gentle physical activity with mindfulness, yoga has increasingly been explored as a strategy for enhancing resilience during times of elevated stress. Nadholta et al. in their study showed yoga can reduce the stress. This study found that yoga can have a positive effect on perceived stress and overall well-being among pregnant women. Such interventions may also be applicable in CBRN situations, where stress levels are elevated, thereby potentially ameliorating mental health outcomes(68). This study was methodologically sound and its findings may be applicable to CBRN contexts, though cultural attitudes toward yoga may affect its transferability.

Claudia et al showed that lemon aromatherapy was effective on Pregnancy Anxienty in the third trimester in Covid 19 Pandemic(64). While the results were positive, the small sample size and subjective measures limited the strength of evidence.

Güney et al. in their results cited that meditation and mindfulness could be reduced stress and axiety in pregnant women during Covid-19 pandemic(49). The study supported mindfulness-based interventions, though the lack of baseline comparisons made changes harder to attribute solely to the intervention.

Also, L Salehi et al. in their study showed that enjoying the joy of the pregnancy experience can help improve mental health(55).

Although conceptually appealing, this observation was difficult to measure objectively and lacked empirical validation.In summary, relaxation-based and mindfulness interventions show positive effects, although evidence remains limited by small samples and short-term follow-up.

1. Organizational level:

Organizational-level interventions emphasize strengthening healthcare systems, ensuring continuity of maternal services, and integrating psychosocial support within disaster and pandemic response frameworks.The second main category was the “organizational level”, derived from four subcategories: “Psychosocial and Maternal Health Support Infrastructure and Education”, “Maternal and Child Healthcare services”, “Mental Health Support for Mothers”, “Response and Risk Communication”.

- Acsess to suppport system and education

Ishii et al. using a large longitudinal survey after the Fukushima disaster, found that psychosocial symptoms in pregnant women outweighed physical ones. They emphasized the need for ongoing regional surveys and parenting support to ensure access to care, including counseling through various channels (66). The study’s large, longitudinal design strengthened its findings, though its focus on a regional disaster limits broader applicability. Its emphasis on mental health support highlights the need for adaptable systemic responses in diverse cultural settings.

Masjoudi et al’s study showed that participation of pregnant women in decisions can be helped to their well-being(18). While this study had a moderate sample size, its cross-sectional design limited causal inferences. The finding supports the psychological theory that autonomy enhances well-being, but its implementation depends heavily on organizational culture and policy frameworks.

Ruxandra-Gabriela Cigăran et al. highlighted the need for a multidisciplinary approach in obstetrics during the pandemic, stressing psychological support and protection for pregnant women (60). Though rich in context, the study lacked quantitative validation and showed challenges in integrated care.

The results of study of Ohtsuru et al. found that prioritizing continuity of care in evacuation zones was crucial during disasters (19). This study had a limited geographic scope but high relevance to disaster settings. Its findings align with broader disaster management principles but require more empirical data to quantify outcomes on maternal mental health.

Maeda et al. emphasized the need for governmental financial support to sustain care networks and called for teamwork among professionals and better screening tools for psychiatric risks such as suicidal behavior (31). While their mixed-methods study offered rich insights, it did not deeply explore the political and economic challenges to implementing such recommendations.

Furthermore, Masaharu Maeda, Misari Oe, and Yuriko Suzuki emphasized the importance of strong interprofessional collaboration in CBRN contexts and called for more efficient screening tools to detect psychiatric risks, including suicidal behavior(31). Their mixed-methods study offered comprehensive insights, though practical implementation of financial and systemic suggestions may face political and economic barriers that were not fully explored.

Ahmad and Vismara recommended long-term, multicenter cohort studies to establish standardized screening and intervention guidelines for pregnant and postpartum women during COVID-19 (50). This suggestion recognized the limitations of many cross-sectional studies and the need for higher-quality evidence. Their call for multicenter designs could improve generalizability but demands substantial funding and coordination.

Some of the studies were done by Aya Goto et al, M Masjoudi et al, Juul Gouweloos et al, Ohtsuru et al, Ahmad and Laura Vismara, and Lebel et al demonestrated that Social support networks can be involved to help mothers. This network can include family and friends(18, 19, 25, 32, 50, 57). These convergent findings across various designs and contexts strengthen the evidence for social support's role. However, the extent and nature of support varied, and some studies lacked standardized measures, limiting comparisons.

Ohtsuru, Koichi et al cited that it should be Coordinated efforts among healthcare professionals, government, and community organizations(19). This organizational-level recommendation aligns with disaster response literature but requires operational frameworks often missing in resource-limited settings.

Masjoudi, et al showed that the pregnant women need to psychological counseling and social support program for pregnant. Furthermore, they cited that plylicymakers should be aware of effective factors on psychological health and self-care of pregnant women.in COVID 19(18). The policy implications are strong, yet the study's cross-sectional nature and regional focus restrict direct application elsewhere.

As noted by Juul Gouweloos et al., .psychosocial care in CBRN settings requires targeted communication, structured preparation, and long-term local support (25). This comprehensive study used mixed methods and longitudinal follow-up, providing high-quality evidence but implementing such extensive programs could be challenging in low-resource settings. Overall, these studies highlight that coordinated support systems and multidisciplinary care are essential for protecting maternal mental health during crises.

- Maternal and Child Healthcare services

The key cods in the results of articles and their authores included: The key codes in the results of the articles and their authors included the following: Masjoudi et al. emphasized the importance of prenatal and postnatal care. They highlighted the need to provide services such as birth planning information, postpartum care, COVID-19 vaccination, and parental guidance during the pandemic (18). While the study provided practical suggestions, its localized and small sample limits generalizability.

Goto et al and [Ishii](https://pubmed.ncbi.nlm.nih.gov/?term=Ishii+K&cauthor_id=28330401) et al. belived that psychologocal child health examinations should be conducted. These studies added a vital perspective on child mental health, which indirectly supports maternal well-being. However, both had limited sample sizes and did not provide detailed protocols for integrating psychological assessments in routine care(32, 66). In conclusion, sustaining routine maternal and child healthcare services remains a critical organizational priority during disasters.

- Mental Health Support for Mothers

Several studies stressed the importance of targeted mental health support for pregnant women and mothers affected by disasters and pandemics. Goto et al. suggested that telephone counseling and psychological support by midwives and nurses could help mothers affected by nuclear accidents. They investigated maternal self-confidence, focusing on its risk factors and connection to maternal depression, finding that evacuation and radiation concerns are strongly associated with depressive symptoms but not with reduced maternal confidence, despite a significant relationship between the two outcomes(32). The cross-sectional design limits causal inference.

Goto et al. in another study emphasized prioritizing mental health support for mothers during nuclear disaster responses and addressing regional mental health disparities, especially for those with disrupted maternity care.n their study stated that Addressing symptoms of maternal depression should be concidered in Fukushima Nuclear Accident(52). This study provided critical insight into systemic gaps in disaster response. Yet, it lacked specific intervention models for addressing regional disparities.

Masjoudi et al. found that self-care can be effective in controlling psychological disorder symptoms among mothers (18). While the study linked self-care and mental health, it did not deeply explore which specific self-care behaviors yield the most benefit, reducing its practical utility.

Lebel et al. found that Higher depression and anxiety symptoms were linked to greater concerns about COVID-19 risks, lack of prenatal care, relationship strain, and social isolation(57). This study had a large sample size and used validated scales, which was a strength. However, it relied on self-reported data, which might have caused some bias.

Ito et al. emphasized the importance of long-term mental health support to address persistent psychological impacts effectively(67). This longitudinal perspective was valuable, but the generalizability was limited by the specific cultural and environmental context in which the study was conducted.

Goto et al. emphasized empowering mothers as a key strategy for post-disaster recovery(52). The study could be strengthened by detailing how empowerment was assessed or promoted. Taken together, the literature supports the need for long-term and accessible maternal mental health interventions, although specific models require further development.

- Disaster Response and Risk Communication

Effective disaster response and risk communication interventions aim to reduce anxiety by providing accurate information, countering misinformation, and strengthening public trust. Key findings, as coded in Table 1, include the following:

Gouweloos et al. examined psychosocial care in CBRN incidents, underscoring the importance of effective public communication and healthcare system preparedness. Their study highlighted distinct challenges in psychosocial support during CBRN events compared to other disasters. Lemyre et al. stated that risk communication should actively counter rumors, correct misinformation, and address social stigma(56). Furthermore, Masjoudi et al. emphasized the importance of regularly monitoring mothers' health, including providing detailed information about symptoms, diagnosis, treatment, and complications, as well as addressing psychological factors such as stress and anxiety(18). Their emphasis on the psychological impact of misinformation is highly relevant but largely theoretical without a tested communication framework.

Maeda et al. and Sena et al. stated that managing disturbing information in the media is necessary during disasters(53, 58). Both studies fall short in offering concrete policy or technological solutions.

Furthermore, studies conducted by Ito et al., Masjoudi et al., Aksoy et al., Lemyre et al., Levi et al., Salehi et al., Tom Farrell et al., and Ito et al. emphasize the importance of assessing information needs and providing detailed information about the incident, including the extent of the incident and the individuals at risk(17, 18, 51, 55, 56, 58, 63, 67). This consensus strengthens the case for proactive risk communication but also signals a gap in operational models.

Maeda et al. highlighted the importance of developing risk communication skills to effectively manage crises and build public trust through accurate information(31). Their focus on communication competence adds value, but the study did not evaluate skill-building interventions.

Juul Gouweloos et al ., Lemyre et al., Maeda et al in their studies focused on effective risk communication and tailored preparation requirements(25, 56). Their triangulated findings reinforce the need for audience-specific messaging; however, practical guidance remains sparse.

Ito, et al., focused on parent training in their results and Gouweloos et al emphasized on rescuere training in disasters(17, 25). Training-focused research is often more actionable, but both studies lacked follow-up to assess behavior change or program effectiveness.

Studies by Lebel et al. (57) and Demisi et al. (70) showed that physical activity helps reduce anxiety and depression symptoms in pregnant women.Studies have shown that exercise is effective in reducing symptoms of anxiety and depression in pregnant women. These findings are strongly supported by empirical data and suggest an accessible intervention, although effects may vary by cultural or individual factors.

The COVID-19 pandemic has accelerated the adoption of tele-psychology and technological devices to provide psychological interventions remotely. Research has highlighted the potential benefits of these tools in reducing the negative effects of the pandemic on mental health(71). Telehealth adoption is promising, but more rigorous longitudinal studies are needed to confirm its long-term benefits. Overall, proactive communication and preparedness training are repeatedly identified as key components of psychosocial care during CBRN and pandemic emergencies.

3- Social level:

Social-level interventions focus on community-based support networks, public initiatives, and long-term coordination to enhance resilience among pregnant women during crises.

- Community Initiatives

Gouweloos et al. providing public communication programs, education, support and counseling and psychosocial care and treatment(25). Their approach offered a comprehensive framework, but the study primarily remains at a conceptual level without evaluating the implementation or effectiveness of these interventions.

Akira Ohtsuru, Koichi, and colleagues cited in their results the importance of planning for the evacuation of large and vulnerable populations.

Goto et al. (18), Masjoudi et al. (32), Ohtsuru et al.(19), Aksoy et al.(58), Lemyre et al.(56), Levi et al.(51), and Lebel et al.(57) emphasized the importance of both general social support—such as emotional, informational, and practical help from family, friends, institutions, or society at large—and local social support, which includes assistance from neighbors, local organizations, or nearby networks.

The broad agreement across studies has strengthened the evidence base for multifaceted social support. However, the heterogeneity in methodologies and outcome measures continues to pose challenges for comparing effectiveness across different contexts.

Implementing Public anti-stigma campaigns was another code that stated by Maeda et al.(31). The absence of intervention trials or metrics for stigma reduction makes it difficult to assess the success of such campaigns.

Ohtsuru et al. described that panning for public health services is a necessity and also, after the nuclear accident, pregnant women in the evacuation zone needed to change their clinics/hospitals and many of them received inadequate antenatal care(19).

The study offered critical insight into structural failures post-disaster. However, it did not explore long-term maternal or neonatal outcomes resulting from disrupted care.

Furthermore, Ishii et al. cited that pregnant women in Fukushima prefecture feared for their children's health, with midwives and public health nurses providing support by phone or email to respondents who were thought to need support for anxiety or health problems(65). This study relied heavily on qualitative self-reporting, which limited generalizability.

Gouweloos et al. emphesized on community support after disasters. They cited that planning for psychosocial care after disasters to create a sense of security, peace, self-efficacy and connection with the community(25). These findings were conceptually strong, though no validated tools were used to measure community connection or recovery outcomes.

Ravaldi et al Emphasis on the importance of reliable support programs and interregional cooperation. Forms of support include interregional cooperation, assessment of individual needs by medical professionals, provision of detailed information to reduce radiation anxiety, routine care for pregnant and lactating mothers(62). The study stood out for proposing multi-level interventions, but its practical feasibility across regions remained uncertain.

Salehi et al. highlight the need to address barriers such as travel restrictions to health centers, proposing community-based interventions and remote healthcare solutions as effective strategies to mitigate anxiety and promote mental well-being during crises(55). This was a particularly actionable recommendation with high relevance during pandemics or radiological events, though long-term outcome data is still lacking. In summary, community initiatives and social support networks play a central role in reducing maternal distress, although effectiveness varies across contexts.

- Long-term Planning and Coordination

This sub categories emerged from the following results that cited by the reserchers:

In a study by Gouweloos et al., the importance of planning for sustained support and resources for mothers and families affected by crises is emphasized(25). Their recommendation is strategic but would benefit from a longitudinal evaluation to assess how sustained interventions affect mental health outcomes.

Maeda et al. describe the need to address mental health stigma, noting that unlike natural disasters, the event led to both psychological distress symptoms and social consequences such as weakened community ties. They called for long-term mental health care networks and community-based campaigns to reduce both general psychiatric and radiation-related stigma (53). This study had holistic view addressing both psychological and social dimensions. However, the evidence remains mostly descriptive, with limited intervention data or measurable stigma reduction.

Ito et al. address the informational needs of mothers regarding radiation exposure and emphasize the importance of managing health risks associated with such exposure(17). This study could be strengthened with quantitative data on how communication reduced anxiety or improved behaviors. Overall, long-term planning and sustained psychosocial care networks are essential to address both psychological distress and social consequences following CBRN-related disasters.
