# Supplementary material for: Psychological Interventions for Pregnant Women in Chemical, Biological, Radiological, and Nuclear Incidents: A Systematic Review
Source: Health Sci Rep. 2026 Jun 2;9(6):e72217. doi: 10.1002/hsr2.72217 (PMC13239930; doi:10.1002/hsr2.72217)
Supplement: Supplementary file 3 — Supporting File 3 [file HSR2-9-e72217-s001.docx]

|  | **Title** | **Author** | **Year/Cou** | **Design** | **Sampl** | **Psychological interventions designed for pregnant women.** |
| --- | --- | --- | --- | --- | --- | --- |
| 1 | The Fukushima Nuclear Accident Affected Mothers’ Depression but Not Maternal Confidence(31) | Aya Goto et al | Apan/ 2017 | Cross-sectional surveys | 13109 | - Providing telephone counseling by midwives and nurses  - Attention to general risk factors for maternal self-confidence and maternal depression symptoms.  - Prioritizing individual and general mental health support for mothers with babies  - Emphasis on maternal and child health services.  - Child health examination  - Strengthening support for mental health of mothers  -Social support for mothers |
| 2 | fukushima Mothers’ Concerns and Associated Factors After the Fukushima Nuclear Power Plant Disaster: Analysis of Qualitative Data From the Fukushima Health Management Survey, 2011 to 2013(17) | Ito, Shinya et al | Apan/ 2017 | Qualitative analysis | 12,415 | - Meeting the informational needs of mothers about radiation exposure  - Paying attention to mothers' concerns after the nuclear accident  - Long-term support for mental health and empowerment of mothers after the accident and proper training of parents  - Continuous accurate assessment of mothers' health  - Providing advice and support to parents, regular and accurate assessment of mothers' health |
| 3 | explaining the ­experience of prenatal care and investigating the association between psychological factors with self-care in pregnant women during COVID-19 pandemic: a mixed method study protocol(18) | Marzieh  Masjoudi, et al | Iran / 2020 | A mixed-methods study with a sequential explanatory design | 215 | - Paying attention to psychological factors such as stress and anxiety  - Planning to try and improve prenatal care services  - Providing care services online, over the phone or in in compliance with health protocols  - Psychological counseling and social support program for pregnant women  - Necessary educational program regarding the symptoms, complications and prevention of covid-19  plan for guidance on nutrition, exercise, rest and self-care  - Providing information about birth planning and postpartum care  - Providing information about the covid 19 vaccination for pregnant and lactating women with a doctor's opinion  -Participation of pregnant women in decisions related to their well-being and measures that can be taken to care for them.  - Understanding the experiences and views of pregnant women regarding prenatal care and factors affecting it during disease outbreaks, especially increasing the effectiveness of providing services and meeting women's needs and expectations.  - Policymakers' awareness of effective factors during pregnancy and self-care of pregnant women. |
| 4 | psychosocial care to affected citizens and communities in case of CBRN incidents: A systematic review(6) | Juul Gouweloos et al | Netherlands / 2014 | A systematic literature review | - | - psychosocial care in CBRN incidents requires special attention to risk communication and its specific preparation.  - Psychosocial support for citizens and groups at risk  - Attention to psychosocial care and treatment for citizens and beneficiaries  - Providing public communication programs, education, support and counseling and psychosocial care and treatment  -Emphasis on public communication against danger and crisis, training of rescuers, support and counseling and psychosocial care  - Emphasis on risk communication and specific preparation needs  -Planning for psychosocial care after disasters to create a sense of security, peace, self-efficacy and connection with the community.  - Support local services for several years after the disaster  - Interventions after a CBRN event must be practical and feasible and planned  - Family is a priority  - Control measures by hospitals. |
| 5 | nuclear disasters and health: lessons learned, challenges, and proposal(19) | Akira Ohtsuru, Koichi et al | Japan/ 2015 | The article and evidence-based interventions | - | - planning for the evacuation of large and vulnerable populations  - Planning mental and psychological care and behavioral and social support for refugees by creating coordinated government groups, municipalities, academic and voluntary organizations.  - Planning to provide public health services  - Planning and coordinating the sending of scientific messages based on the accumulated evidence of past nuclear and atomic bombing incidents by health care professionals with the aim of increasing public understanding of the effects of the incidents.  - Planning and insisting on the continuity of medical care in the facilities of the evacuation zone and surrounding areas  Medical care for vulnerable residents  - Increasing the awareness and preparedness of society to deal with the dangers of radiation  - Comprehensive and coordinated planning to deal with chemical and nuclear accidents |
| 6 | Effects of COVID-19 anxiety and obsession on fear of childbirth in high-risk pregnancy during the pandemic in Turkey(53) | Sena D. Aksoy et al | Turkey/ between March 2021 and March 2022. | a quantitative descriptive study | 326 | -Access to health care and disturbing information in the media  -Increasing social support  -Engaging in more health-promoting behaviors  - -Access to health care |
| 7 | A Psychosocial Risk Assessment and Management Framework  to Enhance Response to CBRN Terrorism Threats and Attacks(51) | Louise Lemyre | Canada / 2005 | Rewive | - | - Psychosocial risk assessment and management framework with the aim of identifying psychological problems and providing appropriate interventions to reduce negative effects and increase resilience and adaptive responses in the face of threats and attacks  - Risk communication  includes risk communication strategies to dispel rumors, correct misinformation, and address social stigma issues  - Education  - Social support  - Professional advice |
| 8 | The Psychological Impact of COVID-19 Pandemic on Women’s Mental Health during Pregnancy: A Rapid Evidence Review(62) | Monica Ahmad and Laura Vismara | December 2020 to January 2021. | Review | - | - Regular physical activity  - Receive social support from family, friends and partners  - Computer screening aimed at facilitating the expression of emotions |
| 9 | Psychological Investigation on Pregnant Women during the Outbreak of COVID-19 (57) | Shaoqi Chen | China / 2020 | Cross-sectional study | 1160 | Self-entertainment activities, such as listening to music or reading books.  chat with family members or friends.  search for solutions online.  consult obstetricians, and some seek advice from professional psychological counselors. |
| 10 | The Psychological Impact of the COVID-19 Pandemic on Pregnant Women (56) | Ruxandra-Gabriela Cigăran et al | Romania/ May and October 2020 | cross-sectional survey | 557 | - The negative impact of restrictions and changes in maternity care  - Emphasis on mental support to improve the mental health of pregnant women and prevent negative consequences of pregnancy  - Psychological support to improve their mental health to prevent negative pregnancy outcomes  - The increasing need for a multidisciplinary approach to pregnant women, obstetrics and gynecology, due to the consequences of the pandemic, with the new responsibility of medical staff to ensure psychological comfort for pregnant women. |
| 11 | Psychosocial effects of the Fukushima disaster and current tasks: Differences between natural and nuclear disasters (30) | Masaharu Maeda, Misari Oe, and Yuriko Suzuki | Japan / 2018 | Review | - | - Development of more efficient screening systems and tools to identify individuals at risk for psychiatric disorders and related issues, especially suicidal behaviors.  - Planning to foster good cooperation with different types of health professionals  - Development of risk communication skills  - Implementation of public anti-stigma campaigns with the participation of mass media  - Planning to provide intensive psychological care for aid workers, especially government employees  - Planning to secure government financial support to maintain and develop the current care network. |
| 12 | Anxiety during pregnancy after the Chernobyl accident as related to obstetric outcome (58) | Ragnar Levi et al | Sweden/ 1989 | prospective study | 86 | - Special care, such as focused attention and support from maternity health care workers, including objective information about perceived health risks  - Social support from family and friends  - The interaction of psychological factors and midwifery parameters together. |
| 13 | Immediate effects of the Fukushima nuclear power plant disaster on depressive symptoms among mothers with infants: a prefectural-wide cross-sectional study from the Fukushima Health Management Survey (46) | Aya Goto et al | Japan / 2010-2011 | prefectural wide cross sectional study | 8196 | - The priority of mental health support for mothers with babies in the early stages of response to nuclear accidents  - Planning to improve the level of mental health of mothers with babies in the acute phase of response to a nuclear disaster  - In the immediate aftermath of a disaster, responses targeting mental health must build on existing systems.  - Providing strategic support to parents  - Addressing regional variations in negative mental health outcomes, particularly for those with interruptions in their maternity care.  - Attention to known risk factors for mental health problems in the post-disaster setting |
| 14 | Postpartum mental health of mothers in fukushima: insights from the fukushima health management survey’s 8-year trends (63) | Kayoko Ishii et al | Japan / 2021 | a survey | 24444 | - Provision of consecutive telephone advice and support by midwives and public health nurses  - Forms of support include interregional cooperation, assessment of individual needs by medical professionals, provision of detailed information to reduce radiation anxiety, routine care for pregnant and lactating mothers.  - Conduct surveys, quickly review responses and identify people in need of support |
| 15 | Pregnant women voice their concerns and birth expectations during the COVID-19 pandemic in Italy (59) | Claudia Ravaldi et al | Italy / 2019 | cross sectional | (200) | - Emphasizing the importance of reliable support programs including health care workers and birth attendants  - Planning and emphasizing taking into account the feelings of women and their special needs to create a respectful alliance and empower women  - Women with a psychological history need special attention and care, because their worries, fears and anxieties are particularly relevant. |
| 16 | The relationship among fear and anxiety of COVID-19, pregnancy experience, and mental health disorder in pregnant women: A structural equation model  (49) | Leili  Salehi et al | Iran / 2020 | cross-sectional | 222 | - Attention and importance to provide information about increasing mothers' awareness of the corona virus, its risk factors and its effect on the fetus and newborn  - Paying attention to travel restrictions to health centers to reduce anxiety  - Planning and emphasizing to reduce the worries of corona and pregnancy, as well as the feeling of enjoying the joy caused by the experience of pregnancy during the pandemic |
| 17 | The impact of the COVID-19 pandemic on the perinatal mental health of women (60) | Tom Farrell et al | Qatar / 2020 | cross- sectional survey | 288 | - Planning and emphasizing coping strategies for women in Qatar regarding the epidemic and precautionary measures during pregnancy  - Attention and importance to planning the help of maternity staff and televised public health information  - public health education initiatives in the current and future health crisis  - Attention and importance to health care in reducing anxiety and stress  - The attention of specialists to the provision of health care services by the organization |
| 18 | Elevated depression and anxiety symptoms among pregnant individuals during the COVID-19 pandemic (52) | Catherine Lebel et al | Canada/ 2020 | Survay | 1987 | - Planning for perceived social support from partner, friends, family and others  - social support  - physical activity  - Paying attention to changes in maternity care, women's negative feelings due to restrictions, especially those related to pregnancy care  - Planning and attention to create preventive measures during the epidemic |
| 19 | Mental Health Consequences and Social Issues After the Fukushima Disaster (47) | Masaharu  Maeda et al | Japan | Review | - | - Providing telephone counseling with the mental health support team every year since the disaster for evacuees at risk and psychiatric disorders such as PTSD or depression  - Providing mental health intervention programs consisting of psychiatrists, social workers, clinical psychologists, nurses and occupational therapists.  - The importance of providing new facilities in an effort to maintain good relationships and communication with other mental health professionals  - Attention to suicide prevention programs  - Planning to remove public stigma towards psychiatric treatments as well as "radiation stigma" |
| 20 | Pregnancy and Birth Survey of the Fukushima Health Management Survey (64) | [Kayoko Ishii](https://pubmed.ncbi.nlm.nih.gov/?term=Ishii+K&cauthor_id=28330401) at all | Japan/ 2011 to 2014/2017 | Survey | 60860 | - Planning to support parents  - Long-term mental health care and coping with stigma.  - Providing Maternal and Child Health Handbooks  - Assessing the physical and mental health of mothers, and offering adequate parenting support (telephone counseling) for those in need |
| 21 | Overview of the pregnancy and birth survey section of the  Fukushima Health Management Survey:  Focus on mothers’ anxieties about radioactive exposure (65) | Shinya Ito et al | Japan/ 2018 | Survey | 310 | - Providing long-term mental health support  - Providing adequate information about the health effects of radiation  - Support for mothers who are concerned about stigma. |
| 22 | The effect of giving lemon aromatherapy and health education on pregnancy anxienty covid 19 pandemic at dungingi health center and kota barat health center the city of gorontalo (61) | Juli Gladis Claudia at al | Indonesia/ 2020 | Quasi Experiment | 60 | - Lemon aromatherapy  - health education |
| 23 | Potential benefits of Yoga in pregnancy related complications during the COVID-19 pandemic and implications for working women(66) | Pooja Nadholta at al | 2020 | literature review | - | - Using yogaas a stress-reducing techniques  - Receive routine pregnancy care |
| 24 | Effectiveness of progressive muscle relaxation technique on anxiety caused by Covid-19 in pregnant women: A randomized clinical trial (50) | Mojgan Zendehdel at al | Iran/ 2020 | randomized clinical trial | 126 | - Progressive muscle relaxation technique  - exercise programs |
| 25 | Effect of the Mindfulness-Based Stress Reduction program on stress, anxiety, and childbirth fear in pregnant women diagnosed with COVID-19 (54) | Esra Güney at al | Turkey/ 2020 | randomized clinical trial | 84 | - The online Mindfulness-Based Stress Reduction (MBSR) program.  - consultancy and support to address any additional questions.  - meditation techniques from Mindfulness Therapy, including body and breath exercises, mindfulness movement, and three-minute breathing techniques. |

Table 2. Categorization of psychological support interventions for pregnant women based on the included studies
